# Unveiling Rare Hemoglobinopathies: Hematologic Characterization of Double Heterozygous Hb D and Hb E With Beta-Thalassemia—A Case Report

**DOI:** 10.1155/crh/8375604

**Published:** 2025-07-06

**Authors:** Aiman Mahmood Minhas, Hadia Eiman, Javed Iqbal, Ayisha Imran, A. S. Chughtai

**Affiliations:** ^1^Department of Hematology, Chughtai Institute of Pathology, Lahore, Punjab, Pakistan; ^2^Department of Medicine, Holy Family Hospital, Rawalpindi, Punjab, Pakistan; ^3^Department of Nursing and Midwifery Education, Hamad Medical Corporation, Doha, Qatar; ^4^CEO of Chughtai Healthcare, Chughtai Institute of Pathology, Lahore, Punjab, Pakistan

**Keywords:** beta-Thalassemia (D017086), capillary (D019075), electrophoresis, hemoglobin D (C032001), hemoglobin E (D006446), hemoglobinopathies (D006453), thalassemia (D013789)

## Abstract

**Background:** Hemoglobinopathies are genetic disorders of hemoglobin, with over 700 variants. Common types include beta-thalassemia, Hb S, Hb E, Hb D, and Hb C, and their prevalence is increasing, especially in developing regions of sub-Saharan Africa and Asia. Pakistan, located in the “thalassemia belt,” has a high rate of these disorders, with beta-thalassemia being the most common. Genetic combinations, including compound heterozygosity, can lead to unpredictable and severe clinical outcomes. Understanding such rare presentations can aid in more accurate diagnosis, better management strategies, and a deeper insight into the genetic diversity of hemoglobinopathies. It also emphasizes the importance of genetic screening in populations with high hemoglobinopathy prevalence, such as Pakistan, to improve patient outcomes.

**Case Presentation:** A one-year-old girl from consanguineous parents in Multan presented with fatigue, feeding difficulties, and severe growth retardation. She had a history of severe anemia requiring a transfusion at 6 months. Examination revealed pallor and mild hepatosplenomegaly. Hemoglobin analysis showed severe anemia (Hb 5.3 g/dL) and a dimorphic blood picture, with electrophoresis indicating compound heterozygosity for Hb D and Hb E, predominated by Hb D. Her father was a compound heterozygote for Hb E and beta-thalassemia. However, the mother was heterozygous for Hb D. Genetic profiling was not completed due to resource limitations, but the family was counseled on consanguinity risks.

**Conclusion:** Given the rising prevalence of uncommon severe hemoglobinopathies in Pakistan and existing resource limitations, targeted screening in high-risk districts and enhanced patient counseling are essential to mitigate the disease burden and improve diagnostic and management strategies.

## 1. Introduction

Hemoglobinopathies is an umbrella term comprising a diverse group of recessive genetic disorders of hemoglobin, both structural and functional [1]. The number of hemoglobin variants described so far exceeds 700 different types. They are broadly classified as thalassemia syndromes and structural hemoglobin variants, the most common being Hb S, Hb E, and Hb C [2]. The prevalence of these diseases is currently on the rise in the world's developing regions, particularly in sub-Saharan Africa and Asia [3]. About 300,000 to 400,000 babies worldwide are born with serious hemoglobin disorders with about 90% emanating from resource-limited countries [4]. According to WHO, this amounts to about 7% of the world's population being victims of these heritable diseases [5].

Pakistan, a developing nation, strategically lies in what is called the “thalassemia belt” and, thereby, has a high prevalence of hemoglobinopathies [6]. According to a Lahore, Punjab province survey, 9.7% of the observed samples had some hemoglobinopathy [7]. After beta-thalassemia major, Hb D trait, sickle/beta-thalassemia, sickle cell disease, Hb E trait, and sickle cell trait, beta-thalassemia minor was the most common ailment in the Pakistani population. The rare forms at the other end of the spectrum are delta/beta-thalassemia, Hb E homozygous, Hb D homozygous, and Hb H disease [8].

Although these hemoglobinopathies occur commonly in homozygous forms, the inheritance of two different variants of a specific gene cluster is rare. These individuals are said to have compound heterozygosity [9]. Such presentations often have more severe and disastrous consequences than their homozygous counterparts. For instance, the clinically nonsignificant forms of Hb D and Hb E combined with beta-thalassemia can have a variable and probably more intense presentation than either of these entities alone [10].

Hb D has about 15 different variants, the most frequent being Hemoglobin D-Punjab. All present with the same amino acid substitution: glutamic acid is replaced by glutamine at position 121 of the beta-globin chain (E121Q, or β121 Glu ⟶ Gln) [11]. In contrast, Hb E possesses a point mutation at position 26 of the beta-globin chain where lysine takes the place of glutamic acid. (E26K or β26 Glu ⟶ Lys) [12]. Here, we present a unique case harboring compound heterozygosity of beta-thalassemia and structural hemoglobin variants while simultaneously co-inheriting Hb D and Hb E forms.

This uncommon genetic combination can result in unpredictable and potentially severe clinical outcomes, making it essential to recognize such rare presentations. By understanding these atypical cases, healthcare providers can enhance diagnostic accuracy and develop more effective management approaches. Here, we present a severe case with co-inheritance of Hb D and Hb E which highlights the genetic diversity of hemoglobinopathies and the importance of genetic screening, particularly in high-prevalence regions like Pakistan, to improve early detection and patient care outcomes.

## 2. Case Presentation

A one-year-old girl, the only child and the product of a consanguineous marriage presented to a local basic health unit within the suburbs of Multan with complaints of fatigue, lethargy, drowsiness, and feeding difficulties. The parents were concerned regarding the well-being of their child as she did not appear as active as other infants her age. Her weight was less than the third percentile, indicating severe growth retardation. The family also reported delays in the achievement of milestones. She was born via spontaneous vaginal delivery at home by a midwife. Her birth was uncomplicated. Vaccination status was unknown. Feeding history included exclusive breastfeeding. There was no difficulty in latching but the baby has had decreased milk intake since around 4–5 months. Past medical history suggested a diagnosis of severe anemia for which she was transfused with 1 pint of Red Cell Concentrate at the age of 6 months.

Family history revealed the death of one of her family members on her paternal side at an early age. The specific disorder and relation to the patient were unknown, as the family belonged to a poor socioeconomic background. According to the examination findings, the patient was lying in her mother's lap, appearing drowsy. Her hydration was adequate but her nutrition status was below satisfactory. She had failed to thrive and was markedly pale, with pallor spanning the entire body surface. There was also mild hepatosplenomegaly on abdominal examination. The rest of the examination was not significant.

Her blood sample was received at Chughtai Lab, Lahore with the request to perform hemoglobin electrophoresis due to the lack of essential resources and equipment in the basic health unit of the rural area. We received 4 mL of venous blood in two ethylenediamine tetraacetic acid (EDTA) vials. One EDTA sample was run on an automated hematology analyzer Sysmex XN 1000 to obtain the patient's hematological parameters including her hemoglobin level, total leucocyte count, platelet counts, total RBC count, MCV, MCH, and MCHC. Her CBC findings showed Hb 5.3 g/dL, TLC 8.7 × 10^3^/μL, platelet 319 × 10^3^/μL, MCV 49 fL, MCH 11.4 pg, MCHC 23.2 g/dL and RBC count 4.6 × 10^6^/μL as indicated in Table 1.

A dimorphic blood image was observed in her peripheral smear, containing macrocytes, polychromasia, hypochromic microcytic red blood cells with prominent anisopoikilocytosis, and a few fragmented and nucleated red blood cells. The complete RBC morphology is given in Table 2.

**Figure 1:**
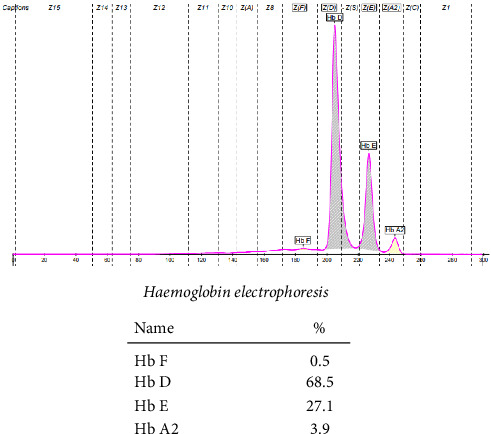
Hemoglobin electrophoresis of the patient.

The patient's hemoglobin electrophoresis was performed on SEBIA Capillary 2 Flex piercing using capillary electrophoresis. The results showed some striking features. The patient had a compound heterozygous state indicating the presence of both Hb D and Hb E with dominance of Hb D as illustrated in Figure 1.

To understand the inheritance pattern we requested the blood samples of her parents for hemoglobin electrophoresis. Using the same techniques described earlier, the following results were obtained. Her father's CBC showed Hb 14.3 g/dL, MCV 82 fL, MCH 26.9 pg, and MCHC 32.8 g/dL as shown in Table 1. The red blood cells in his peripheral smear were normocytic and normochromic.

**Figure 2:**
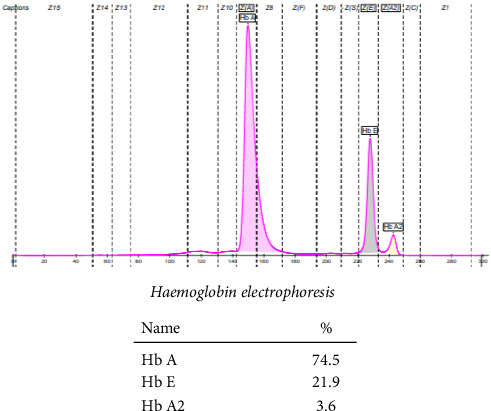
Hemoglobin electrophoresis of the father.

Hemoglobin electrophoresis showed the presence of Hb D with co-inheritance of the beta-thalassemia trait, evidenced by decreased HbA and a rise in HbA2 above the normal level. With these findings, the father was labeled as a compound heterozygote for Hb E/beta-thalassemia as evidenced in Figure 2.

Blood sample from the mother was also evaluated through rigorous testing. Her CBC showed Hb 12.8 g/dL, MCV 79 fL, MCH 25 pg, and MCHC 31.9 g/dL. The details of other blood indices are delineated in Table 1. Furthermore, the peripheral smear she had revealed normocytic normochromic red blood cells.

**Figure 3:**
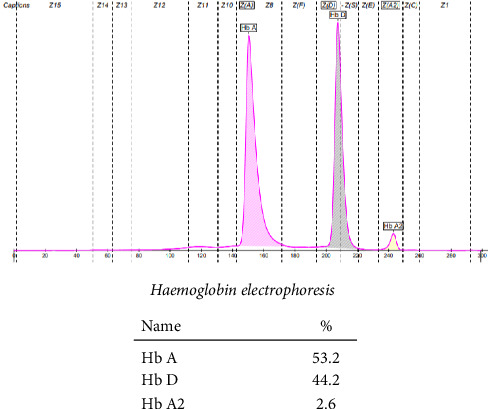
Hemoglobin electrophoresis of the mother.

Hemoglobin electrophoresis showed another insidious finding. Her blood sample showed an abundance of Hb D (44.2%) and Hb A (53.2%). She was labeled as heterozygous for Hb D, delineated in Figure 3.

**Figure 4:**
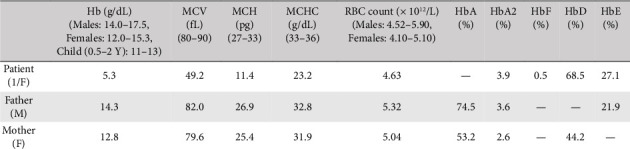
Pedigree of family with HbD/E–β Thalassemia double heterozygosity.

Based on the cumulative findings, we suspected the patient to be compound heterozygous for Hb D and Hb E with coinheritance of beta-thalassemia. For confirmation the HPLC was performed on patient's sample and it confirmed the compound heterozygosity. To further confirm our diagnosis molecular studies were performed in some other laboratory. According to the report provided by the patient's attendant the testing was performed using Sanger DNA sequencing. The results showed IVSI-5 mutation. In such cases, beta-thalassemia minor presents clinically as beta-thalassemia major, in concert with the severity of presentation and anemia.  Figure 4 sheds light on the pedigree analysis of the patient's immediate household.

Treatment with regular blood transfusions and iron chelation was advised. Bone marrow transplant was kept in plan whenever a suitable donor became available. Genetic profiling was advised to shed more light on the inheritance patterns of these hemoglobin subtypes in the family. However, this process remained uncompleted due to a scarcity of resources and problems with transportation in the area where the patient resided. As the family was illiterate and came from a poor socioeconomic class, they failed to comply with regular follow-ups and further testing. However, the family was counseled, elaborating on the risks and highlighting the associations with consanguineous marriages, in an attempt to halt the progression of such devastating hemoglobinopathies.

## 3. Discussion

The significance of laboratory diagnosis of hemoglobinopathies, including thalassemia and structural hemoglobin disorders, is profound. This has led to increased use of testing modalities to screen for these disorders as early as the prenatal and antenatal period [13]. While these diseases are on the rise worldwide, Pakistan faces a particularly high and serious challenge as it lies at the center point of these disorders, along with other countries in Southeast Asia [14]. Beta-thalassemia, in both major and carrier forms, is the most common hemoglobinopathy in Pakistan, along with Hb S, Hb E, and Hb D-Punjab [15].

Hemoglobin variant Hb E is commonly found in Bangladesh and other South Asian countries, as well as in the eastern section of the subcontinent. With the co-inheritance of Hb E, a particularly severe form of beta-thalassemia occurs, which might present as one of the common hemoglobinopathies in these areas [16]. Hb D is another recurrent structural variant in the South Asian population, particularly affecting India, Pakistan, Iran, Iraq, and others along the eastern coastline. It may also occur in heterozygous forms associated with either iron deficiency anemia or, on the other extreme, co-inheritance of beta-thalassemia [17].

The distinct incidences of hemoglobin D/beta-thalassemia and hemoglobin E/beta-thalassemia in Pakistan, were 1.4% each in the studied population of Karachi [18]. Isolated Hb D is clinically silent and represents a relatively mild hemoglobin variant. However, when co-inherited with beta-thalassemia, it can lead to significant pathology [19]. Hb E alone also does not result in a significant clinical problem until it is co-inherited with other thalassemia syndromes, resulting in devastating sequelae [20]. However, a combination of these hemoglobinopathies, harboring compound heterozygosity for Hb D and Hb E along with inheritance of beta-thalassemia presents a complex clinical picture as indicated in this case report.

Our case study presented a unique finding of compound heterozygosity of Hb D and Hb E with co-inheritance of beta-thalassemia. The patient's history of early anemia, failure to thrive, and need for transfusion indicated a severe hemoglobinopathy at play. The patient's history of early anemia, failure to thrive, and need for transfusion suggestive of complex interplay of genetic factors and iron deficiency might contribute to the clinical phenotypes. The same clinical phenotype has been observed in this patient from India. No molecular analysis was done to determine the heterozygous state of Hb D in that study [21].

Another case of a 2-year-old boy was reported in the same region of Bihar, India whose clinical picture reflected severe microcytic anemia with evidence of hemolysis and jaundice. Ultrasonography revealed mild splenomegaly along with free fluid in the peritoneum. Laboratory investigations rendered him a case of Hb E/beta-thalassemia, with the father being heterozygous HbA/E and the mother carrying the beta-thalassemia trait [22].

Beta-thalassemia and Hb D co-inheritance were also identified in a Thai child aged seven who had mild anemia and microcytosis at presentation for her annual checkup. Capillary electrophoresis showed the presence of Hb D-Punjab. Detailed molecular analysis for detecting the beta-thalassemia mutation gave positive results. Interestingly, the Hb A2 measured on capillary electrophoresis proved to be a reliable differentiating factor between Hb D homozygous and Hb D/beta-thalassemia compound heterozygous [23]. This case study delineated the vast prevalence of hemoglobinopathies in the susceptible regions of Southeast Asia, compounding the thalassemia belt.

Out of 21 individuals with Hb D disease in the studied population of Saudi Arabia, Hb D co-inheritance with beta-thalassemia was observed in two cases. The disease manifested in moderate hemolytic anemia with the domination of Hb D. The combined presence of beta-thalassemia with Hb D was conferred to be responsible for the clinical presentation of these individuals lying on the severe side of the spectrum for Hb D disease [24]. This highlights the far-fetched reach of hemoglobinopathies outside the thalassemia belt, suggesting a worldwide genetic problem.

Pakistan is a developing nation with a scarcity of resources, a lack of awareness, and efficient patient follow-up strategies, all required to combat such complex health conditions. The increased occurrence of hemoglobinopathies raises significant concern over the present state of health in the country. This calls for screening to be done in high-risk districts in the prenatal and antenatal period along with premarital and pre-pregnancy counseling to reduce the burden of disease. Further, standardized testing and detailed clinical assessments could identify a larger cohort of patients presenting with similar co-inheritance of various hemoglobin subtypes. This can lead to the development of treatment strategies for efficient disease control.

## 4. Conclusion

In conclusion, this rare case harboring compound heterozygosity of beta-thalassemia with Hb D and Hb E reflects the importance of recognizing uncommon genetic combinations that can result in unpredictable and severe clinical outcomes. The rising prevalence of hemoglobinopathies, particularly in regions like Pakistan and Southeast Asia, presents a significant health challenge. Co-inheritance of several hemoglobin variations, including beta-thalassemia, Hb D, and Hb E, complicates clinical presentations, highlighting the need for early and accurate diagnosis through prenatal, antenatal, and premarital screening. Laboratory diagnostics are crucial in identifying these disorders, guiding patient management, and genetic counseling. In resource-limited settings like Pakistan, enhanced awareness, standardized testing, and better healthcare infrastructure are essential to mitigate the burden of these complex genetic diseases.

## Figures and Tables

**Table 1:** Complete blood count of the patient, father, and mother.

Components	Patient	Father	Mother
WBC (10^3^/μL)	8.73	7.02	8.88
RBC (10^6^/μL)	4.63	5.32	5.04
HB (g/dL)	5.3	14.3	12.8
HCT (%)	22.8	43.6	40.1
MCV (fL)	49.2	82.0	79.6
MCH (pg)	11.4	26.9	25.4
MCHC (g/dL)	23.2	32.8	31.9
Platelet (10^3^/μL)	319	331	517
RDW-CV	29.6	14.1	14.9

**Table 2:** RBC morphology of the patient.

RBC morphology	
Hypochromia	++
Microcytosis	++
Macrocytosis	+
Anisocytosis	++
Poikilocytosis	++
Target cells	+
Polychromasia	+
Fragmented RBCs	+

## Data Availability

The data that support the findings of this study are available from the corresponding author upon reasonable request.
